# Pre-selection against a lethal recessive allele in breeding schemes with optimum-contribution selection or truncation selection

**DOI:** 10.1186/s12711-021-00669-4

**Published:** 2021-09-22

**Authors:** Line Hjortø, Mark Henryon, Huiming Liu, Peer Berg, Jørn Rind Thomasen, Anders Christian Sørensen

**Affiliations:** 1 Center for Quantitative Genetics and Genomics, Aarhus University, Blichers Allé 20, 8830 Tjele, Denmark; 2 SEGES, Agro Food Park 15, 8200 Aarhus N, Denmark; 3 Danish Pig Research Centre, SEGES, Axeltorv 3, 1609 Copenhagen V, Denmark; 4 School of Agriculture and Environment, University of Western Australia, 35 Stirling Highway, Crawley, WA 6009 Australia; 5 Department of Animal and Aquaculture Sciences, Norwegian University of Life Sciences, 1432 Ås, Norway; 6 VikingGenetics, Ebeltoftvej 16, 8960 Randers SØ, Denmark

## Abstract

**Background:**

We tested the hypothesis that breeding schemes with a pre-selection step, in which carriers of a lethal recessive allele (LRA) were culled, and with optimum-contribution selection (OCS) reduce the frequency of a LRA, control rate of inbreeding, and realise as much genetic gain as breeding schemes without a pre-selection step.

**Methods:**

We used stochastic simulation to estimate true genetic gain realised at a 0.01 rate of true inbreeding (ΔF_true_) by breeding schemes that combined one of four pre-selection strategies with one of three selection strategies. The four pre-selection strategies were: (1) no carriers culled, (2) male carriers culled, (3) female carriers culled, and (4) all carriers culled. Carrier-status was known prior to selection. The three selection strategies were: (1) OCS in which $\Delta {\text{F}}_{{{\text{true}}}}$ was predicted and controlled using pedigree relationships (POCS), (2) OCS in which $\Delta {\text{F}}_{{{\text{true}}}}$ was predicted and controlled using genomic relationships (GOCS), and (3) truncation selection of parents. All combinations of pre-selection strategies and selection strategies were tested for three starting frequencies of the LRA (0.05, 0.10, and 0.15) and two linkage statuses with the locus that has the LRA being on a chromosome with or without loci affecting the breeding goal trait. The breeding schemes were simulated for 10 discrete generations (*t* = 1, …, 10). In all breeding schemes, ΔF_true_ was calibrated to be 0.01 per generation in generations *t* = 4, …, 10. Each breeding scheme was replicated 100 times.

**Results:**

We found no significant difference in true genetic gain from generations *t* = 4, …, 10 between breeding schemes with or without pre-selection within selection strategy. POCS and GOCS schemes realised similar true genetic gains from generations *t* = 4, …, 10. POCS and GOCS schemes realised 12% more true genetic gain from generations *t* = 4, …, 10 than truncation selection schemes.

**Conclusions:**

We advocate for OCS schemes with pre-selection against the LRA that cause animal suffering and high costs. At LRA frequencies of 0.10 or lower, OCS schemes in which male carriers are culled reduce the frequency of LRA, control rate of inbreeding, and realise no significant reduction in true genetic gain compared to OCS schemes without pre-selection against LRA.

## Background

Frequencies of lethal recessive alleles - hereafter referred to as LRA - can increase in livestock populations for two reasons. First, LRA can hitchhike to high frequencies when they are linked to alleles that improve traits under selection [1]. Second, LRA can drift to high frequencies when small numbers of sires make large genetic contributions to the next generation [1]. Lethal recessive alleles at high frequencies are undesirable both ethically and economically, particularly when they cause death at or after birth. Most breeding schemes aim at maximising genetic gain ($\Delta {\text{G}}$) at acceptable rates of inbreeding ($\Delta {\text{F}}$). However, in a population with carriers of LRA, we need breeding strategies that support three aims: (1) decrease the frequency of LRA, (2) control $\Delta {\text{F}}$ at acceptable levels, and (3) realise as much $\Delta {\text{G}}$ as breeding schemes without a strategy to reduce the frequency of LRA. Strategies, which are traditionally used in breeding programs to decrease the frequency of LRA, select against them in two steps. First, male carriers are excluded from the breeding program in a pre-selection step. Second, breeding animals are selected by truncation selection from the remaining candidates [2]. These strategies are effective in reducing the frequency and the expression of LRA. However, they have two drawbacks. First, the realised ΔG may decrease because carriers with high genetic merit can be excluded from the breeding program by pre-selection [3]. Second, it may cause bottlenecks and higher $\Delta {\text{F}}$ [4]. Therefore, current strategies may reduce the frequency of LRA, but they are ineffective at controlling $\Delta {\text{F}}$ at acceptable levels and at realising as much $\Delta {\text{G}}$ as breeding schemes without a strategy to reduce the frequency of LRA.

We think it is possible to define a breeding strategy that supports all three aims, and the most efficient method to reduce the frequency of a LRA is to cull some or all carriers in a pre-selection step. However, intense culling of carriers creates disarray in ancestral genetic contributions, with parents that carried the LRA having a decreased number of offspring among the selection candidates. Therefore, these parents will have a lower probability of having superior offspring selected under truncation selection, so the parents’ genetic contributions will be reduced. A strategy that may enable us to control $\Delta {\text{F}}$ and maintain $\Delta {\text{G}}$ while reducing the frequency of LRA is to combine pre-selection with optimum-contribution selection (OCS) of parents. Optimum-contribution selection is a selection strategy that could be useful here because it maximises $\Delta {\text{G}}$ at a given $\Delta {\text{F}}$ [5, 6]. It realises higher $\Delta {\text{G}}$ for a given $\Delta {\text{F}}$ than truncation selection in breeding schemes without a pre-selection step [6]. It could also maximise $\Delta {\text{G}}$ at a given $\Delta {\text{F}}$ with a pre-selection step by better managing the genetic contributions of selection candidates and their ancestors to the next generation. Our reasoning is founded on the theory of genetic contributions [7]. The theory shows that the optimal relationship between true Mendelian-sampling term and long-term genetic contribution maximises $\Delta {\text{G}}$ at a fixed $\Delta {\text{F}}$. The optimal relationship is a threshold linear relationship. If the true Mendelian sampling term of an individual is larger than the threshold, the long-term genetic contribution of that individual is positive and linearly related to the true Mendelian sampling term. If the true Mendelian sampling term is less than the threshold, the contribution is zero. In practical breeding schemes, the true Mendelian sampling terms are not known and estimated Mendelian sampling terms are used instead. The use of estimated Mendelian sampling terms and the fact that genetic contributions of mated animals are not independent cause deviations from the optimal relationship between true Mendelian sampling term and long-term genetic contribution [8]. Pre-selection also generates deviations from the optimal relationship by changing genetic contributions of some parents of the selection candidates. The squared deviations from the optimal relationship are minimised when OCS is used. Optimum-contribution selection allocates genetic contributions of each selection candidate to the next generation and thereby rearranges genetic contributions of their ancestors. This rearranging aims at reducing any deviations including those introduced by pre-selection. Thus, we hypothesize that breeding schemes with a pre-selection step and OCS reduce the frequency of the LRA, control ΔF, and realise as much $\Delta {\text{G}}$ as breeding schemes without a pre-selection step.

## Methods

### Design

We used stochastic simulation to estimate true genetic gain ($\Delta {\text{G}}_{{{\text{true}}}}$) realised at a 0.01 rate of true inbreeding ($\Delta {\text{F}}_{{{\text{true}}}}$) by breeding schemes that combined one of four pre-selection strategies with one of three selection strategies. The four pre-selection strategies were: (1) no carriers culled, (2) male carriers culled, (3) female carriers culled, and (4) all carriers culled. Carrier-status was known prior to selection. The three selection strategies were: (1) OCS in which $\Delta {\text{F}}_{{{\text{true}}}}$ was predicted and controlled using pedigree relationships (POCS), (2) OCS in which $\Delta {\text{F}}_{{{\text{true}}}}$ was predicted and controlled using genomic relationships (GOCS), and (3) truncation selection of parents. All combinations of the pre-selection strategies and the selection strategies were tested for three starting frequencies of the LRA and two options for which the locus with the LRA was on a chromosome with or without loci affecting the breeding goal trait, hereafter referred to as linkage status. The simulation consisted of three parts: a founder population, a base population, and a population under selective breeding. Selection was for a single trait with a heritability of 0.20 and was controlled by 2320 biallelic quantitative trait loci (QTL). The QTL were randomly distributed across a 30-M genome that consisted of 30 pairs of autosomes, each 100 cM long. The number of QTL was 80 for all pairs of chromosomes except one chromosome, i.e. chromosome 1, which had no QTL. Chromosome 1 was used to test linkage status. Each pair of chromosomes had 1400 markers and the genome included a total of 42,000 biallelic markers. These markers were randomly distributed along the genome and in linkage disequilibrium (LD) with the QTL. Genomic estimated breeding values (GEBV) were used as predictions of genetic merit. In total, 6000 identical-by-descent (IBD) loci were placed evenly along the genome of animals in the base populations. Unique alleles at these loci were used to calculate $\Delta {\text{F}}_{{{\text{true}}}}$. Generations were discrete. All animals were genotyped at birth and all female selection candidates after the base population were phenotyped prior to selection. Parents were mated randomly in all generations. The breeding schemes were simulated for 10 generations ($t$ = 1, …, 10). In all breeding schemes, $\Delta {\text{F}}_{{{\text{true}}}}$ was calibrated to be 0.01 per generation in generations $t$ = 4, …, 10 averaged across replicates. Each combination of pre-selection strategy, selection strategy, frequency, and linkage status was replicated 100 times.

### Selection strategies

#### Optimum-contribution selection

The methods that we used to carry out POCS and GOCS are similar to the methods used by Henryon et al. [9, 10]. In short, POCS allocated matings to selection candidates in generations $t$ = 2, …, 10 on the basis of a quadratic function, ${\text{U}}_{t}$, that was maximised with respect to ${\mathbf{c}}$:

$${\text{U}}_{t} \left( {\mathbf{c}} \right) = {\mathbf{c^{\prime}g}} - \omega {\mathbf{c}}^{\prime}{\mathbf{Ac}},$$

where ${\mathbf{c}}$ is a $n$ vector of genetic contributions to the next generation and the number of matings distributed to each candidate is a linear function of these contributions, $n$ is the number of animals in the population traced back from the candidates in generation $t$ to the base population, ${\mathbf{g}}$ is a $n$ vector of GEBV, $\omega$ is a penalty applied to the expected average relationship of the next generation, and ${\mathbf{A}}$ is a $n \times n$ pedigree-relationship matrix. Elements of ${\mathbf{c}}$ were constrained to 0 ≤ ${\text{c}}_{i}$ ≤ 0.5 ($i$ = 1, …, $n$) with ${\text{c}}_{i}$ = 0 for animals that were not selection candidates in generation $t$. We calibrated $\omega$ in each breeding scheme to realise a $\Delta {\text{F}}_{{{\text{true}}}}$ of 0.01 from generations $t$ = 4, …, 10 as an average across replicates. As a result, an average of 35 sires were selected per generation. The number of selected sires could differ between generations and replicates. In GOCS schemes, we substituted ${\mathbf{A}}$ with a $n \times n$ genomic-relationship matrix, ${\mathbf{G}}$, which was obtained by VanRaden’s first method [11] and traced back to the base population. On average, 32 sires were selected per generation.

#### Truncation selection

Males with the highest GEBV were selected as sires. The average number of sires used to achieve a $\Delta {\text{F}}_{{{\text{true}}}}$ of 0.01 from generations $t$ = 4, …, 10 was 61 sires per generation. The number of selected males varied between schemes but was the same within schemes in all replicates and all generations.

### Generations −3000 to −1: founder population

We used the founder population with a high level of LD as in Thomasen et al. [12]. The LD pattern resembled the LD observed in Nordic Holstein and Danish Jersey dairy cattle. Offspring inherited alleles from their parents at QTL and marker loci following Mendel’s rules of inheritance allowing for mutations and recombinations. Generation $t$ =  − 1 of the founder population consisted of 25 males and 25 females. All 42,000 markers and 2320 QTL segregated in these animals. Additive allelic effects for all QTL were randomly sampled from an exponential distribution. The effects were scaled such that the additive genetic variance was 1. For more information on the founder population see Thomasen et al. [12].

We selected one QTL on either chromosome 1 or 2 that matched the desired frequency of the LRA in all schemes. The starting frequency of the LRA was 0.05, 0.10, or 0.15. The LRA showed complete penetrance and caused death among newborn animals when it was present in the homozygous state. This meant that animals that were homozygous for the LRA could never be selection candidates. The LRA did not have an additive effect on the trait under selection, but it was either unlinked or linked to QTL with additive effects on the trait under selection according to its position on chromosome 1 or 2, respectively.

Chromosomes from the 50 animals in generation $t$ =  − 1 were pooled: 30 pools of 100 chromosomes. Each pool consisted of 50 chromosome pairs of the $i$-th chromosome ($i\,$ = 1 … 30) from 50 founder animals.

### Generation 0: base population

The base population consisted of 100 females and 11 males. For each replicate, the base population was sampled from the chromosome pools from generation $t$ = − 1. Each base animal received two copies at random from each pool. For each animal in the base population, the true breeding value (TBV) was obtained by summing the allelic effects at each QTL. Sampling caused variation in the frequency of the LRA. The standard deviations of the means were on average 0.0142, 0.0195, and 0.0238, when the starting frequencies of the LRA were 0.05, 0.10, and 0.15. After sampling, each base animal was assigned two unique alleles at each IBD locus. IBD alleles could be traced back from each descendant to the base animal from which it was derived. The assignment of IBD alleles was done as in Henryon et al. [10]. The animals in the base population were assumed to be unrelated and non-inbred based on pedigree and alleles at IBD loci. In the base population and in all subsequent generations, QTL, markers, and IBD loci were sampled according to Mendel’s rules of inheritance. The animals in the base population were genotyped but not phenotyped.

### Generation 1: random selection in the base population

Generation 1 consisted of 500 offspring of 10 randomly selected males and 100 females from the base population, i.e. each male was mated to ten random females and each female produced five offspring. The number of males in the base population was randomly reduced from 11 to 10 to construct a genomic-relationship matrix that was positive definite. In generation 1 and in all subsequent generations, a phenotype was realized for each female selection candidate prior to prediction of GEBV and selection. The phenotype was calculated as the sum of the TBV and the residual environmental value. The TBV was obtained in the same way as in the base population. The residual environmental value was sampled from a normal distribution with a mean of 0 and a residual environmental variance of 4.0. The residual environmental variance was constant over generations, whereas the genetic variance and the heritability decreased across generations of selection due to fixation and the Bulmer effect [13].

### Generations 2 to 10: selective breeding

Selection candidates in generations $t$ = 1, …, 9 were pre-selected based on carrier-status. Subsequently, parents of the animals in generations $t$ = 2, …, 10 were selected by POCS, GOCS or truncation selection. One hundred females were selected per generation among approximately 250 female selection candidates. The selected females were randomly mated with the selected males and produced five offspring each, resulting in 100 full-sib families and 500 offspring. Each offspring had an equal probability of being a male or a female.

### Genomic prediction

Breeding values were predicted prior to selection using a best linear unbiased prediction (BLUP) animal model:

$${\mathbf{y}} = {\mathbf{Xb}} + {\mathbf{Zu}} + {\mathbf{e}},$$

where ${\mathbf{y}}$ is a vector of phenotypic observations, ${\mathbf{b}}$ is a vector of fixed generation effects, ${\mathbf{u}}$ is a vector of random animal effects, ${\mathbf{e}}$ is a vector of random residual effects, ${\mathbf{X}}$ and ${\mathbf{Z}}$ are incidence matrices relating phenotypic observations to fixed effects and random animal effects. The following (co)variance structure was used to predict breeding values:

$$\left( {\begin{array}{*{20}c} {\mathbf{a}} \\ {\mathbf{e}} \\ \end{array} } \right)\sim N\left( {{\mathbf{0}};\left[ {\begin{array}{*{20}c} {{\mathbf{G}}{\upsigma }_{{\text{a}}}^{2} } & 0 \\ 0 & {{\mathbf{I}}{\upsigma }_{{\text{e}}}^{2} } \\ \end{array} } \right]} \right),$$

where the genomic relationship matrix ${\mathbf{G}}$ is as described previously, and ${\mathbf{I}}$ is an identity matrix. The variances, ${\upsigma }_{{\text{a}}}^{2}$ and ${\upsigma }_{{\text{e}}}^{2}$, were 1 and 4.

### IBD loci

Each of the animals had 6000 IBD loci that were evenly distributed along the genome. The animals in the base population carried two unique alleles at each of the IBD loci. The IBD alleles were used to calculate $\Delta {\text{F}}_{{{\text{true}}}}$. The mean coefficient of true inbreeding for animals born in generation $t$ ($t$ = 1, …, 10; ${\text{F}}_{{{\text{IBD}}_{t} }}$) was calculated as ${\text{F}}_{{{\text{IBD}}_{t} }} = \frac{1}{{n_{t} n_{{{\text{IBD}}}} }}\mathop \sum \limits_{i = 1}^{{n_{t} }} \mathop \sum \limits_{j = 1}^{{n_{{{\text{IBD}}}} }} {\updelta }_{ij}$, where $n_{t}$ is the number of animals born in generation $t$ ($t$ = 1, …, 10), $n_{{{\text{IBD}}}}$ is the number of IBD loci, and ${\updelta }_{ij}$ is the IBD status of animal $i$ ($i$ = 1, …, $n_{t}$) at IBD locus $j$ ($j$ = 1, …, $n_{{{\text{IBD}}}}$). ${\updelta }_{ij}$ was equal to 1 if animal $i$ was homozygous at IBD locus $j$, and 0 otherwise. The IBD alleles were not used for prediction and selection.

### Data analyses

The results are presented for two time periods: (1) results in the first period show the consequences of the first round of pre-selection and selection, and (2) results in the second period show the consequences of pre-selection and selection approaching equilibrium. Thus, we considered six response parameters: $\Delta {\text{G}}_{{{\text{true}}1 - 2}}$, $\Delta {\text{G}}_{{{\text{true}}4 - 10}}$, $\Delta {\text{F}}_{{{\text{true}}1 - 2}}$, and $\Delta {\text{F}}_{{{\text{true}}4 - 10}}$, and the proportion of replicates where the frequency of the LRA increased in the two time periods. True genetic gain in generations $t$ = 1, 2 and ΔG_true4-10_ were calculated as linear regressions of ${\text{G}}_{t}$ on $t$ for each replicate, where ${\text{G}}_{t}$ is the average TBV of animals born in generations $t$ ($t$ = 1, 2 and $t$ = 4, …, 10). Subsequently, ${ }\Delta {\text{G}}_{{{\text{true}}1 - 2}}$ and $\Delta {\text{G}}_{{{\text{true}}4 - 10}}$ were averaged across replicates within each breeding scheme. Rate of true inbreeding in generations $t$ = 1, 2 and $\Delta {\text{F}}_{{{\text{true}}4 - 10}}$ were calculated as $1 - {\text{exp}}\left( {\upbeta } \right)$ within each replicate, where ${\upbeta }$ is a linear regression of ${\text{ln}}(1 - {\text{F}}_{{{\text{IBD}}_{t} }}$) on $t$ ($t$ = 1, 2 and $t$ = 4, …, 10). This transformation was performed because ${\text{ln}}(1 - {\text{F}}_{{{\text{IBD}}_{t} }}$), not ${\text{F}}_{{{\text{IBD}}}}$, is linear over time. Subsequently, $\Delta {\text{F}}_{{{\text{true}}1 - 2}}$ and $\Delta {\text{F}}_{{{\text{true}}4 - 10}}$ were averaged across replicates within each breeding scheme.

We analysed $\Delta {\text{G}}_{{{\text{true}}1 - 2}}$, $\Delta {\text{G}}_{{{\text{true}}4 - 10}}$, $\Delta {\text{F}}_{{{\text{true}}1 - 2}}$, and $\Delta {\text{F}}_{{{\text{true}}4 - 10}}$, and the proportion of replicates where the frequency of the LRA increased in the two time periods with a four-factor ANOVA model to determine which factors could be excluded from the model. The model included the following factors: pre-selection strategy, selection strategy, starting frequency of the LRA, linkage status, and all possible interactions between them. We reduced the model sequentially using *F*-tests. For each response parameter, the reduced model was used to estimate least-squares means. Differences between levels of factors were tested using Tukey’s method for adjusting p values under multiple testing [14]. We used 0.05 as the level of significance. The tables presented in the paper represent the significant effects on the response parameters averaged across non-significant effects. Within a sample of scenarios, the frequencies of the LRA were averaged within generations and across replicates and plotted as a function of generation.

### Software

We used the program ADAM to carry out the simulations [15], the program DMU6 to predict genomic breeding values [16], the program EVA to carry out OCS [17], and R statistical software to analyse the output of the ADAM simulations [18].

## Results

### True genetic gain

Selection strategy was the only factor that explained variation in $\Delta {\text{G}}_{{{\text{true}}4 - 10}}$. Hence, we found no significant difference in $\Delta {\text{G}}_{{{\text{true}}4 - 10}}$ between breeding schemes with or without pre-selection within selection strategy. POCS and GOCS schemes realised similar $\Delta {\text{G}}_{{{\text{true}}4 - 10}}$. POCS and GOCS schemes realised 12% more $\Delta {\text{G}}_{{{\text{true}}4 - 10}}$ than truncation selection schemes (Table 1). Selection strategy also explained variation in $\Delta {\text{G}}_{{{\text{true}}1 - 2}}$ (Table 2). However, GOCS outperformed both POCS and truncation selection schemes.

**Table 1:** Least-squares means for true genetic gain in generations ${t}$ = 4, …, 10 ($\Delta {\text{G}}_{{{\text{true}}4 - 10}}$) realised by POCS, GOCS, and truncation selection at a 0.01 $\Delta {\text{F}}_{{{\text{true}}4 - 10}}$ in a breeding scheme with genomic prediction

Selection strategy	$\Delta {\text{G}}_{{{\text{true}}4 - 10}}$
POCS	0.528^a^
GOCS	0.530^a^
Truncation selection	0.473^b^

Means with the same superscripted letter are not significantly different from each other (*p* < 0.05)

**Table 2:** Least-squares means for true genetic gain in generation ${t}$ = 2 ($\Delta {\text{G}}_{{{\text{true}}1 - 2}}$) realised by POCS, GOCS, and truncation selection at a 0.01 $\Delta {\text{F}}_{{{\text{true}}4 - 10}}$ in a breeding scheme with genomic prediction

Selection strategy	$\Delta {\text{G}}_{{{\text{true}}1 - 2}}$
POCS	0.494^b^
GOCS	0.515^a^
Truncation selection	0.496^b^

Means with the same superscripted letter are not significantly different from each other (*p* < 0.05)

When the starting frequency of the LRA was 0.05, all $\Delta {\text{G}}_{{{\text{true}}1 - 2}}$ across pre-selection strategies were similar (Table 3). Schemes in which carriers of a single sex were culled realised no significant reduction in $\Delta {\text{G}}_{{{\text{true}}1 - 2}}$ compared to schemes without pre-selection when the starting frequency of the LRA was 0.10. When the starting frequency of the LRA was 0.15, culling carriers reduced $\Delta {\text{G}}_{{{\text{true}}1 - 2}}$ by up to 18% compared to no carriers being culled and mostly so when all carriers were culled. When the starting frequency of the LRA was 0.05, $\Delta {\text{G}}_{{{\text{true}}1 - 2}}$ across linkage status were similar (Table 4). At higher frequencies, the pattern according to linkage status was inconsistent.

**Table 3:** Least-squares means for true genetic gain in generation ${t}$ = 2 according to pre-selection strategy and starting frequency of the lethal recessive allele at a 0.01 $\Delta {\text{F}}_{{{\text{true}}4 - 10}}$ in a breeding scheme with genomic prediction

Pre-selection strategy	Starting frequency of the lethal recessive allele		
	0.05	0.10	0.15
No carriers culled	0.529^a^	0.527^ab^	0.529^a^
Male carriers culled	0.523^ab^	0.513^ab^	0.500^b^
Female carriers culled	0.516^ab^	0.501^b^	0.470^c^
All carriers culled	0.507^ab^	0.468^c^	0.435^d^

Means with the same superscripted letter are not significantly different from each other (*p* < 0 .05)

**Table 4:** Least-squares means for true genetic gain in generation ${t}$ = 2 according to linkage status and starting frequency of the lethal recessive allele at a 0.01 $\Delta {\text{F}}_{{{\text{true}}4 - 10}}$ in a breeding scheme with genomic prediction

Linkage status	Starting frequency of the lethal recessive allele		
	0.05	0.10	0.15
Unlinked	0.518^a^	0.494^b^	0.490^bc^
Linked	0.521^a^	0.510^a^	0.477^c^

Means with the same superscripted letter are not significantly different from each other (*p* < 0.05)

### Rate of true inbreeding

None of the factors in the model or the interactions were significant at the 0.05 level when $\Delta {\text{F}}_{{{\text{true}}4 - 10}}$ was the response variable. This means that the calibration of $\Delta {\text{F}}_{{{\text{true}}4 - 10}}$ was successful. Selecting breeding animals using POCS resulted in slightly lower $\Delta {\text{F}}_{{{\text{true}}1 - 2}}$ than GOCS and considerably lower than truncation selection (Table 5) although the rate of true inbreeding in later generations was the same. Within selection strategy, $\Delta {\text{F}}_{{{\text{true}}1 - 2}}$ tended to be highest with no carriers culled and lowest with female carriers culled. Also, within selection strategy, $\Delta {\text{F}}_{{{\text{true}}1 - 2}}$ was lower at a higher starting frequency of the LRA (Table 6).

**Table 5:** Least-squares means for rate of true inbreeding from generations ${t}$ = 1 to 2 according to pre-selection strategy and selection strategy at a 0.01 $\Delta {\text{F}}_{{{\text{true}}4 - 10}}$ in a breeding scheme with genomic prediction

Pre-selection strategy	Selection strategy		
	POCS	GOCS	Truncation selection
No carriers culled	0.0164^de^	0.0171^d^	0.0247^a^
Male carriers culled	0.0159^ef^	0.0171^d^	0.0237^b^
Female carriers culled	0.0152^f^	0.0166^de^	0.0226^c^
All carriers culled	0.0159^ef^	0.0172^d^	0.0226^c^

Means with the same superscripted letter are not significantly different from each other (*p* < 0.05)

**Table 6:** Least-squares means for rate of true inbreeding from generations ${t}$ = 1 to 2 according to selection strategy and starting frequency of the lethal recessive allele at a 0.01 $\Delta {\text{F}}_{{{\text{true}}4 - 10}}$ in a breeding scheme with genomic prediction

Selection strategy	Starting frequency of the lethal recessive allele		
	0.05	0.10	0.15
POCS	0.0161^d^	0.0157^d^	0.0158^d^
GOCS	0.0172^c^	0.0171^c^	0.0168^c^
Truncation selection	0.0242^a^	0.0233^b^	0.0227^b^

Means with the same superscripted letter are not significantly different from each other (*p* < 0.05)

On average, culling of female carriers led to lower $\Delta {\text{F}}_{{{\text{true}}1 - 2}}$ (Table 7). A higher starting frequency of the LRA leads to a lower $\Delta {\text{F}}_{{{\text{true}}1 - 2}}$, only within culled female carriers. In the other pre-selection strategies, $\Delta {\text{F}}_{{{\text{true}}1 - 2}}$ was similar across starting frequencies.

**Table 7:** Least-squares means for rate of true inbreeding from generations ${t}$ = 1 to 2 according to pre-selection strategy and starting frequency of the lethal recessive allele at a 0.01 $\Delta {\text{F}}_{{{\text{true}}4 - 10}}$ in a breeding scheme with genomic prediction

Pre-selection strategy	Starting frequency of the lethal recessive allele		
	0.05	0.10	0.15
No carriers culled	0.0196^a^	0.0193^ab^	0.0192^abc^
Male carriers culled	0.0194^ab^	0.0187^abc^	0.0186^abc^
Female carriers culled	0.0188^abc^	0.0184^bc^	0.0172^d^
All carriers culled	0.0189^abc^	0.0183^c^	0.0185^bc^

Means with the same superscripted letter are not significantly different from each other (*p* < 0.05)

### Trend of the frequency of the lethal allele

**Fig. 1:**
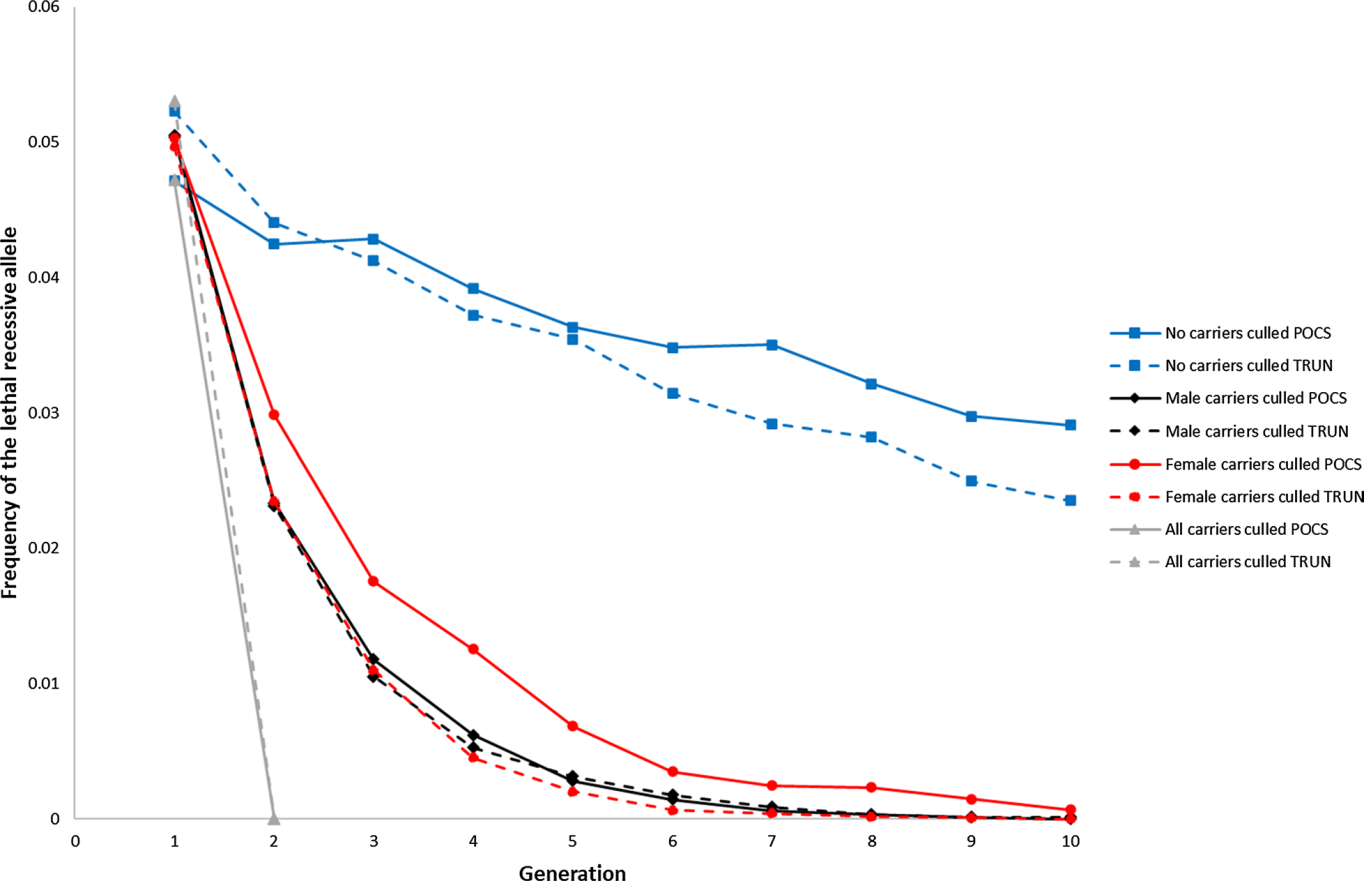
Frequency of the lethal recessive allele (LRA) in generations ${t}$ = 1, …, 10 under optimum-contribution selection using pedigree relationships (POCS, full line) or truncation selection (TRUN, dashed line) when the starting frequency of the LRA was 0.05, the LRA was linked, and no carriers were culled (blue squares), male carriers were culled (black diamonds), female carriers were culled (red circles), or all carriers were culled (grey triangles). Standard deviations of the means were in the range 0 to 0.0414

The frequency of the LRA decreased at a faster rate when carriers were culled in a pre-selection step compared to no carriers being culled (Fig. 1). The frequency was close to zero in generation $t$ = 10 when male or female carriers were culled. When no carriers were culled in a pre-selection step, the frequency of the LRA decreased from approximately 0.05 in generation $t$ = 1 to approximately 0.025 in generation $t$ = 10. This decrease was a result of natural selection because homozygous offspring die. However, in the breeding scheme with no carriers being culled and POCS, the frequency of the LRA increased on average in both generations $t$ = 3 and $t$ = 7 compared to the generations immediately before, as a result of genetic drift. The frequency of the LRA decreased from generation $t$ = 1 to 2 at a slower rate with OCS than truncation selection, whether carriers were culled in a pre-selection step or not. By design, the LRA was completely removed from the population in generation $t$ = 2 when all carriers were culled.

**Fig. 2:**
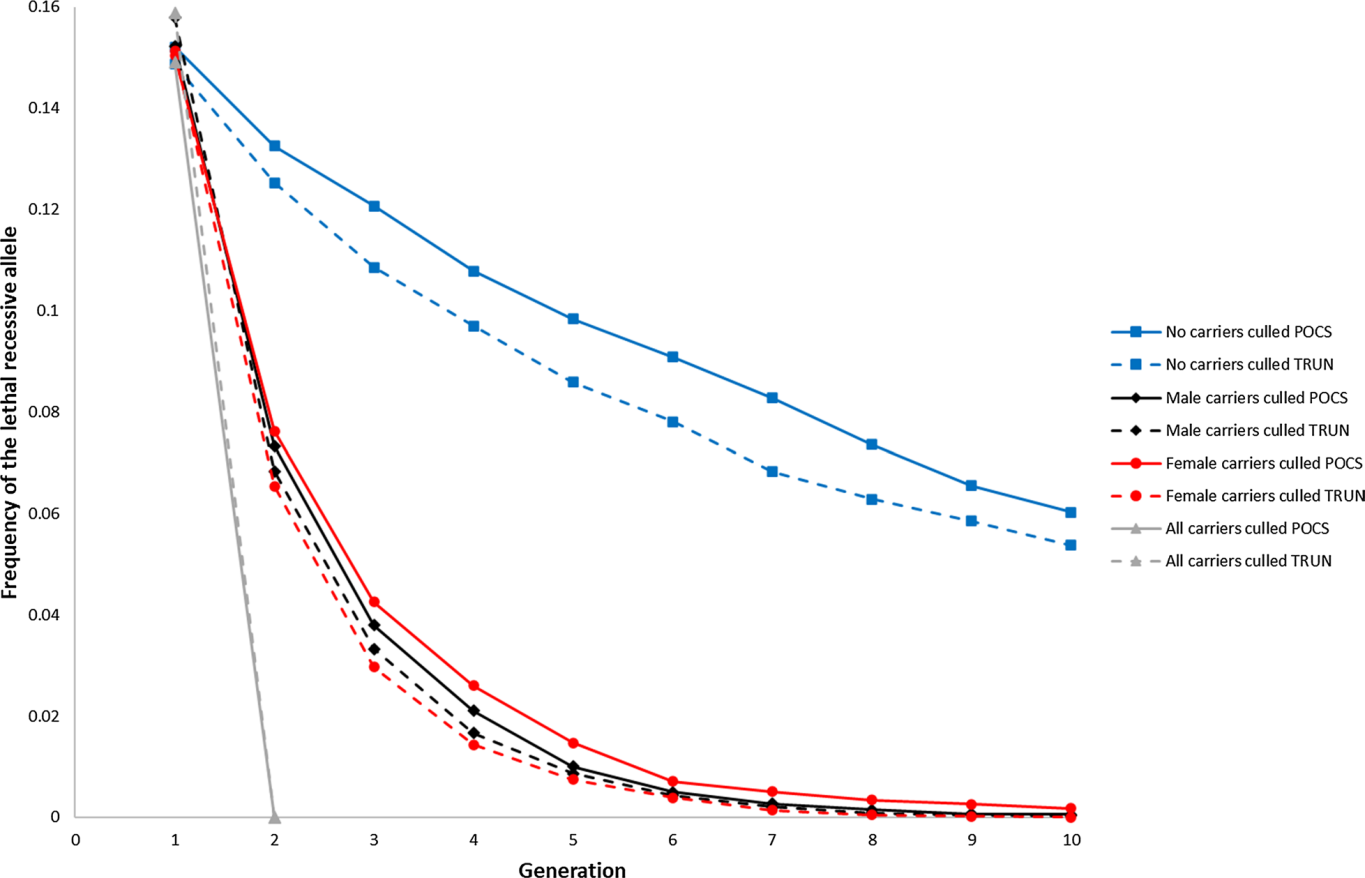
Frequency of the lethal recessive allele (LRA) in generations ${t}$ = 1, …, 10 under optimum-contribution selection using pedigree relationships (POCS, full line) or truncation selection (TRUN, dashed line) when the starting frequency of the LRA was 0.15, the LRA was linked, and no carriers were culled (blue squares), male carriers were culled (black diamonds), female carriers were culled (red circles), or all carriers were culled (grey triangles). Standard deviations of the means were in the range 0 to 0.0534

When no carriers were culled, the average frequency of the LRA did not increase at any time point when its starting frequency was 0.15 (Fig. 2). In addition, the frequency of the LRA decreased at a faster rate when its starting frequency was 0.15 than when it was 0.05 (Figs. 1 and 2). Using a breeding scheme without a pre-selection step and POCS as an example, the frequency of the LRA in generation $t$ = 10 was 40% of its initial value, when its starting frequency was 0.15, but 62%, when it was 0.05. This is because natural selection is relatively stronger than genetic drift when the frequency of the LRA is high. When male or female carriers were culled, the frequency of the LRA was close to zero in generation $t$ = 10 although it was approximately 0.15 in generation $t$ = 1.

When no carriers were culled, the proportion of replicates (> 30%) with the frequency of the LRA increasing from generations $t$ = 1 to 2 was higher than in the other pre-selection strategies (Tables 8 and 9). The proportion of replicates with the frequency of the LRA increasing from generations $t$ = 1 to 2 was lower when male carriers were culled than when female carriers were culled because there were fewer sires than dams and, for that reason, genetic drift played a bigger part when males carried the LRA. When no carriers were culled, POCS resulted in more replicates with the frequency of the LRA increasing from generations $t$ = 1 to 2 than in the other selection strategies, and when female carriers were culled, POCS and GOCS resulted in more replicates with the frequency of the LRA increasing from generations $t$ = 1 to 2 than in truncation selection. Except for the strategy where all carriers were culled, the proportion of replicates with the frequency of the LRA increasing from generations $t$ = 1 to 2 decreased with an increased starting frequency of the LRA (Table 9). When no carriers were culled, this result was consistent with the expectation that natural selection is relatively stronger than genetic drift at higher frequencies.

**Table 8:** Least-squares means for the proportion of replicates where the frequency of the lethal recessive allele increased from generations ${t}$ = 1 to 2 according to pre-selection strategy and selection strategy at a 0.01 $\Delta {\text{F}}_{{{\text{true}}4 - 10}}$ in a breeding scheme with genomic prediction

Pre-selection strategy	Selection strategy		
	POCS	GOCS	Truncation selection
No carriers culled	0.390^a^	0.332^b^	0.337^b^
Male carriers culled	0.027^d^	0.040^d^	0.028^d^
Female carriers culled	0.093^c^	0.092^c^	0.040^d^
All carriers culled	0^d^	0^d^	0^d^

Means with the same superscripted letter are not significantly different from each other (*p* < 0.05)

**Table 9:** Least-squares means for the proportion of replicates where the frequency of the lethal recessive allele increased from generations ${t}$ = 1 to 2 according to pre-selection strategy and starting frequency of the lethal recessive allele at a 0.01 $\Delta {\text{F}}_{{{\text{true}}4 - 10}}$ in a breeding scheme with genomic prediction

Pre-selection strategy	Starting frequency of the lethal recessive allele		
	0.05	0.10	0.15
No carriers culled	0.378^a^	0.365^a^	0.315^b^
Male carriers culled	0.065^de^	0.027^def^	0.003^f^
Female carriers culled	0.132^c^	0.073^d^	0.020^ef^
All carriers culled	0^f^	0^f^	0^f^

Means with the same superscripted letter are not significantly different from each other (*p* < 0.05)

We found no significant differences between the selection strategies for the proportion of replicates with the frequency of the LRA increasing from generations $t$ = 4 to 10. The proportions of replicates with the frequency of the LRA increasing from generations $t$ = 4 to 10 were significantly higher when no carriers were culled than in the other pre-selection strategies (Table 10) and this proportion was lower at a higher starting frequency of the LRA. When carriers were culled, there were very few, if any, replicates with the frequency of the LRA increasing from generations $t$ = 4 to 10, as a proportion of 0.002 corresponds to 1 out of 500 replicates recording an increase in the frequency.

**Table 10:** Least-squares means for the proportion of replicates where the frequency of the lethal recessive allele increased from generations ${t}$ = 4 to 10 according to pre-selection strategy and starting frequency of the lethal recessive allele at a 0.01 $\Delta {\text{F}}_{{{\text{true}}4 - 10}}$ in a breeding scheme with genomic prediction

Pre-selection strategy	Starting frequency of the lethal recessive allele		
	**0.05**	**0.10**	**0.15**
No carriers culled	0.238^a^	0.207^a^	0.152^b^
Male carriers culled	0^c^	0^c^	0.005^c^
Female carriers culled	0.002^c^	0.005^c^	0.007^c^
All carriers culled	0^c^	0^c^	0^c^

Means with the same superscripted letter are not significantly different from each other (*p* < 0.05)

## Discussion

Our results support the hypothesis that it is possible to setup breeding schemes with a pre-selection step and OCS that reduce the frequency of a LRA, control rate of inbreeding, and realise similar $\Delta {\text{G}}_{{{\text{true}}}}$ as breeding schemes without a pre-selection step. This applies to all OCS schemes when the starting frequency of the LRA is low and to OCS schemes that cull carriers of a single sex when the starting frequency of the LRA is intermediate. Contrary to our expectations, we were not able to detect a drop in long-term $\Delta {\text{G}}_{{{\text{true}}}}$ in truncation selection schemes with pre-selection compared to truncation selection schemes without pre-selection. Pre-selection reduced short-term $\Delta {\text{G}}_{{{\text{true}}}}$ when the starting frequency of the LRA was intermediate and all carriers were culled, and when the starting frequency of the LRA was high. The unfavourable consequence of a loss in short-term $\Delta {\text{G}}_{{{\text{true}}}}$ is a genetic gap that cannot be closed because long-term $\Delta {\text{G}}_{{{\text{true}}}}$ in schemes in which all carriers are culled is not larger than long-term $\Delta {\text{G}}_{{{\text{true}}}}$ in schemes in which no carriers are culled. OCS realizes more $\Delta {\text{G}}_{{{\text{true}}}}$ than truncation selection in breeding schemes without a pre-selection step [6]. Our results showed that OCS also realizes more $\Delta {\text{G}}_{{{\text{true}}}}$ than truncation selection in breeding schemes with a pre-selection step. Therefore, a breeding strategy combining pre-selection and OCS should become the method of choice when the aim of the breeding scheme is to reduce the frequency of a LRA, control ${\Delta F}$ and realise as much long-term genetic gain as breeding schemes without a pre-selection step.

Although OCS is still the best selection method, the pre-selection strategy should be defined so that it depends on the frequency of the LRA. We recommend breeding schemes with a pre-selection strategy that culls some or all the carriers if one of the aims of the breeding strategy is to reduce the frequency of a LRA. In this study, we simulated one LRA. However, we believe that our recommendation still applies when the population harbours one LRA with a certain carrier frequency or more LRA with the same total carrier frequency. Natural selection will on average decrease the frequency, but there is a risk that the frequency can actually go up due to genetic drift in any particular replicate. The pre-selection strategy should depend on the frequency of the LRA, so that a less drastic strategy is used when the allele frequency is high, and a more drastic strategy is used when the frequency of the LRA is lower. Our recommendation is supported by Windig et al. [4], who suggest a dynamic breeding strategy so that the recommended selection regime changes as the frequency of the favourable allele increases. Thus, we group our recommendations according to the frequency of the LRA: (1) 0.05 or lower, (2) from 0.05 to 0.10, and (3) higher than 0.10. There are three reasons for this. First, culling carriers of a single sex or culling all carriers supports the three aims in the breeding strategy when the frequency of the LRA is 0.05 or lower. Second, at higher frequencies, pre-selection can also be too drastic, e.g. breeding schemes in which all carriers are culled realise less $\Delta {\text{G}}_{{{\text{true}}}}$ in the short-term than breeding schemes in which no carriers are culled, because culling of carriers creates disarray in genetic contributions. For that reason, we cannot recommend breeding schemes that cull all carriers if the frequency of the LRA ranges from 0.05 to 0.10 and one of the aims of the breeding strategy is to realise just as much $\Delta {\text{G}}_{{{\text{true}}}}$ in breeding schemes with pre-selection as in breeding schemes without pre-selection. Culling carriers of a single sex does not reduce $\Delta {\text{G}}_{{{\text{true}}}}$ or increase $\Delta {\text{F}}_{{{\text{true}}}}$ in the short-term relative to breeding schemes without pre-selection when the frequency of the LRA ranges from 0.05 to 0.10. Our findings are supported by the results of Man et al. [19], who found that schemes in which male carriers are culled reduced the frequency of the unfavourable allele without changing $\Delta {\text{G}}$ and $\Delta {\text{F}}$ significantly compared to schemes in which no carriers are culled. Breeding schemes in which female carriers are culled lead in some cases to a higher proportion of replicates with the frequency of the LRA increasing compared to breeding schemes in which male carriers are culled. In spite of the lower $\Delta {\text{F}}_{{{\text{true}}}}$, this suggests a higher risk for breeding schemes in which female carriers are culled. Therefore, we argue that breeding schemes in which male carriers are culled perform better than breeding schemes with other pre-selection strategies, if the frequency of the LRA ranges from 0.05 to 0.10. Third, if the starting frequency of the LRA is higher than 0.10 then we recommend that a part of the male carriers is culled prior to selection in order to reduce the frequency of the LRA to 0.10. Thus, it seems reasonable to use a less drastic pre-selection strategy when the frequency of the LRA is high and change the breeding strategy to a more drastic pre-selection strategy as its frequency decreases in the population.

There is room for reducing the frequency of some of the LRA that segregate in the population but not all of them. This is supported by our results and the results of Van Eenennaam and Kinghorn [3]. We think it is sensible to distinguish between LRA that cause death at or after birth (e.g. CVM [20], BLAD [21], cholesterol deficiency [22], and a deletion on bovine chromosome 23 in Nordic Red dairy cattle [23]) and LRA that lead to early embryonic death because the LRA in the first group are more likely to cause animal suffering and high costs than those in the second group. The LRA in the first group should be included in the breeding strategy. We propose a dynamic pre-selection strategy where the strategy depends on the frequencies of the LRA. In addition, we propose that the carrier-status of the LRA in the second group are included in a selection index. The expression of the LRA that are still represented in both sexes can be avoided by using a mating strategy that prevents matings between males and females carrying the same LRA, e.g. Cole [24]. However, a mating strategy incapacitates natural selection, i.e. it does not reduce the frequencies of LRA, and it requires that both males and females are genotyped. Georges et al. [25] state that culling carriers of all types of recessive deleterious alleles is not the right approach because the proportion of animals without known recessive deleterious alleles becomes very small as the number of haplotypes harbouring recessive deleterious alleles increases. Thus, breeding organisations should not aim for selecting and marketing breeding animals that do not carry any known recessive deleterious alleles [25]. Georges et al. [25] proposed that carrier-status is included in a selection index. Thus, we agree with the line of reasoning of Georges et al. [25], but we disagree partly with their recommendations. We are aware that index selection theoretically can result in more $\Delta {\text{G}}$ than pre-selection. However, there are two reasons why we prefer a pre-selection strategy for some of the LRA. First, it is very difficult to model the cost of animal suffering properly. Second, there is no guarantee that the frequency of the LRA will decrease when a selection index is used. Another possibility could be to include a third term in the objective function of OCS, e.g. Granleese et al. [26] included cost of female reproductive technologies as a second penalty. The same method could be used to include the cost of LRA. However, our two concerns about index selection also apply for OCS with additional penalties. Our results show that it is possible to set up breeding schemes that reduce the frequency of a LRA, with no compromises on $\Delta {\text{G}}$ or $\Delta {\text{F}}$. Therefore, we advocate for a dynamic breeding strategy in which we divide LRA according to the severity of suffering that the animals experience.

It may be possible to reduce the frequency of a LRA at a faster rate by using a slightly modified ${\mathbf{G}}$-matrix in OCS. In our study, pre-selection reduced the frequency of the LRA within all selection strategies, but OCS slowed this reduction while trying to maintain genetic variation at all loci in the genome. This is undesirable as we would prefer that the healthy wild-type allele becomes fixed at the locus with the LRA. Thus, we want to incapacitate OCS at the locus with the LRA. Roughsedge et al. [27] showed an example of allowing selection to act on a QTL while controlling inbreeding at other regions of the genome by using a relationship matrix based on pedigree and marker information in OCS. Similarly, one could imagine excluding single nucleotide polymorphisms (SNPs) at and near the locus with the LRA from the ${\mathbf{G}}$-matrix in OCS [28]. As a result, OCS cannot counteract the effect of pre-selection on the frequency of the LRA. We could not find significant long-term differences between POCS and GOCS but GOCS has the added feature of potentially differentially weighting the SNP. Therefore, we expect that GOCS combined with clever handling of the LRA will reduce the frequency of the LRA at a faster rate.

## Conclusions

We advocate for OCS schemes with pre-selection against lethal recessive alleles (LRA) that cause animal suffering and high costs. At LRA frequencies of 0.10 or lower, OCS schemes in which male carriers are culled reduce the frequency of LRA, control rate of inbreeding and realise no significant reduction in true genetic gain compared to OCS schemes without pre-selection against LRA. The pre-selection strategy should be less drastic when the frequency of the LRA is high and become more drastic as it decreases in the population. We suggest that the carrier-status of LRA that lead to early embryonic death is included in a selection index. Our suggested strategy for OCS schemes with pre-selection against LRA can easily be combined with a mating strategy that prevents matings between males and females carrying the same LRA. Thus, the expression of those that are still represented in both sexes can be avoided.

## Data Availability

The simulated data sets used and analysed during the current study are available from the corresponding author on reasonable request.
